# Barriers and Enablers to Enacting Child and Youth Related Injury Prevention Legislation in Canada

**DOI:** 10.3390/ijerph13070656

**Published:** 2016-07-07

**Authors:** Linda Rothman, Ian Pike, Kathy Belton, Lise Olsen, Pam Fuselli, Alison Macpherson

**Affiliations:** 1 Faculty of Health-School of Kinesiology & Health Science, Norman Bethune College, York University, 4700 Keele St., Room 337, Toronto, ON M3J 1P3, Canada; alison3@yorku.ca; 2 Department of Pediatrics, University of British Columbia, Vancouver, BC V6T 1Z4, Canada; ipike@cw.bc.ca; 3 BC Injury Research and Prevention Unit, Child and Family Research Institute at BC Children’s Hospital, Room F505, 4480 Oak St., Vancouver, BC V6H 3V4, Canada; 4 Injury Prevention Centre, School of Public Health, University of Alberta, 8308 114 St. NW, Edmonton, AB T6G 2E1, Canada; kathy.belton@ualberta.ca; 5 Faculty of Health and Social Development, School of Nursing, The University of British Columbia, Okanagan, 1147 Research Rd., ART 360, Kelowna, BC V1V 1V7, Canada; lise.olsen@ubc.ca; 6 Knowledge Transfer & Stakeholder Relations, Parachute 150 Eglinton Ave East Suite 300, Toronto, ON M4P 1E8, Canada; pfuselli@parachutecanada.org

**Keywords:** children, youth, legislation, enablers, barriers

## Abstract

Injury prevention policy is crucial for the safety of Canada’s children; however legislation is not adopted uniformly across the country. This study aimed to identify key barriers and enablers to enacting injury prevention legislation. Purposive snowball sampling identified individuals involved in injury prevention throughout Canada. An online survey asked respondents to identify policies that were relevant to them, and whether legislation existed in their province. Respondents rated the importance of barriers or enablers using a 5-point Likert type scale and included open-ended comments. Fifty-seven respondents identified the most common injury topics: bicycle helmets (44, 77%), cell phone-distracted driving (36, 63%), booster seats (28, 49%), ski helmets (24, 42%), and graduated driver’s licensing (21, 37%). The top enablers were research/surveillance, managerial/political support and professional group consultation, with much variability between injury topics. Open-ended comments emphasized the importance of a united opinion as an enabler and barriers included costs of protective equipment and inadequate enforcement of legislation. The results highlighted the importance of strategies that include research, management and community collaboration and that injury prevention topics should be addressed individually as information may be lost if topics are considered together. Findings can inform the process of turning injury prevention evidence into action.

## 1. Introduction

Injury is an important health problem in Canada, particularly for children and youth [1]. Injuries are the leading cause of death for Canadians aged 1–44 [2]. In 2010, almost 16,000 Canadians died, over 230,000 were hospitalized and there were almost 3.5 million emergency department visits due to preventable injury. Injuries can significantly reduce quality of life, with effects ranging from reduced active living to serious, life-long disabilities. Over 60,000 Canadians were left with permanent disabilities due to injury in 2010. The total economic cost of injury was estimated to be 26.9 billion dollars [2].

Injury prevention legislation is an important strategy to reduce the burden of injury. The development of injury prevention legislation is complex, as it may encompass numerous areas outside of the health domain. For example, child injury legislation may originate in product safety (e.g., crib design and child resistant caps), transportation (e.g., child restraints), education (e.g., playground design), or other sectors. Evidence related to the effectiveness of injury prevention legislation has been emerging over the years. For example, a Cochrane review of bicycle helmet laws, reported that helmet use has increased and head injuries have decreased in every study that used comparative designs [3,4]. Similarly, a Cochrane review reported a reduction in motor vehicle collisions among young drivers subsequent to the introduction of legislation requiring graduated driver’s licensing [5]. Evidence also indicates that booster seat laws increase booster seat use and decrease mortality among children 4–8 years [6,7], and that child safety seat laws in general increase child safety seat use and reduce injuries and fatalities [8].

Despite the current level of evidence supporting the effectiveness of injury prevention legislation, laws have not been enacted uniformly across Canada. The factors influencing enactment of legislation may vary by topic. Factors related to the enactment of child injury prevention legislation may also be different from other child health legislation and public policy in general. The purpose of this study was to determine the key barriers and enablers to enacting child and youth injury prevention legislation by topic in Canada.

## 2. Methods

An online survey was designed by an expert panel of six researchers and policy makers from across Canada. This panel represented a broad array of expertise from epidemiology, social sciences research to advocacy and policy implementation. The survey development was further guided by the barriers, facilitators and themes identified in a previous systematic review examining the use of evidence by policymakers (Appendix) [9]. Questions in the current survey were designed based on the barriers and facilitators identified in the review, which were categorized into the following themes depending on content: organizations and resources; contact and collaboration; research and researcher characteristics; policymaker characteristics; and policy characteristics. Access to quality research and the quality of the relationship between the researcher and the policymakers, were identified by the review as being pivotal to the use of evidence by policymakers.

The survey was conducted in the winter/spring of 2015. Purposive snowball sampling identified individuals involved in injury prevention research, practice and policy throughout Canada. Respondents identified provincial-level injury topics they have been involved with, and whether related legislation existed in their province. Respondents rated the importance of potential enablers or barriers to injury legislation by topic using a 5-point Likert type scale (strongly agree, somewhat agree, neutral, somewhat disagree, strongly disagree) and then provided comments. The top five injury topics were identified according to the frequency of responses. The frequency and percentages of “somewhat” and “strongly agree” were tabulated for each question and presented, with the percentages calculated from the total number of responses for each question by topic. Enablers were also examined by topic according to the length of time since legislation enactment in each province. Open-ended comments from across all injury topic areas were examined and common themes identified using thematic analysis [10]. This approach was used to identify, analyze and report patterns across topics as they related to barriers and enablers to legislation. The steps of analysis included coding relevant data segments, and then sorting and reassembling data into themes that provided a more detailed explanation of the barriers and enablers to legislation. The initial thematic analysis was completed by two of the authors and then presented, discussed and agreed upon by all authors. A case study of ski helmet legislation in Nova Scotia is included to highlight enablers to the legislative process. 

All subjects gave their informed consent for inclusion before they participated in the study. The study was conducted in accordance with the Declaration of Helsinki, and Ethics approval for this survey was obtained from York University’s Office of Research Ethics, Human Participants Review Sub-Committee (e2013-154). 

## 3. Results

**Figure 1:**
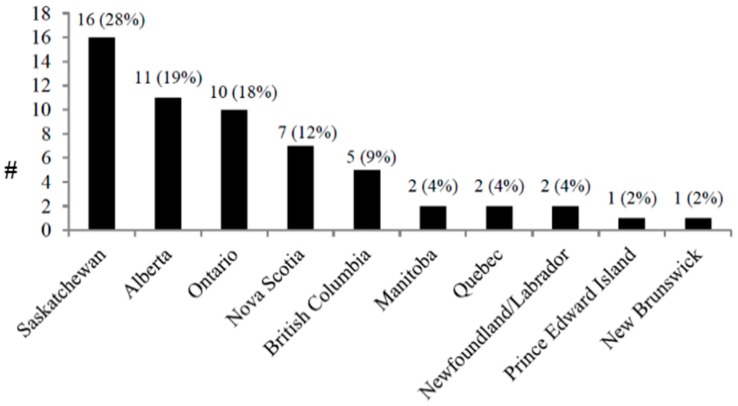
Responses by province (*n* = 67).

There were 57 respondents with representation from all 10 provinces with most from Saskatchewan (16, 28%), Alberta (11, 19%) and Ontario (10, 18%, Figure 1). There were no responses from the Northwest, Nunavut and Yukon Territories. Thirty (53%) worked in health promotion/education/public health, 19 (33%) were policy makers/analysts/consultants, 16 (28%) worked in advocacy, 6 (11%) were researchers and 10 (18%) were unspecified professionals, with overlap among the categories. Thirty-one (54%) of the respondents reported some clinical training. The average years worked in the injury-related profession was 12 years (range 5 months–35 years).

**Figure 2:**
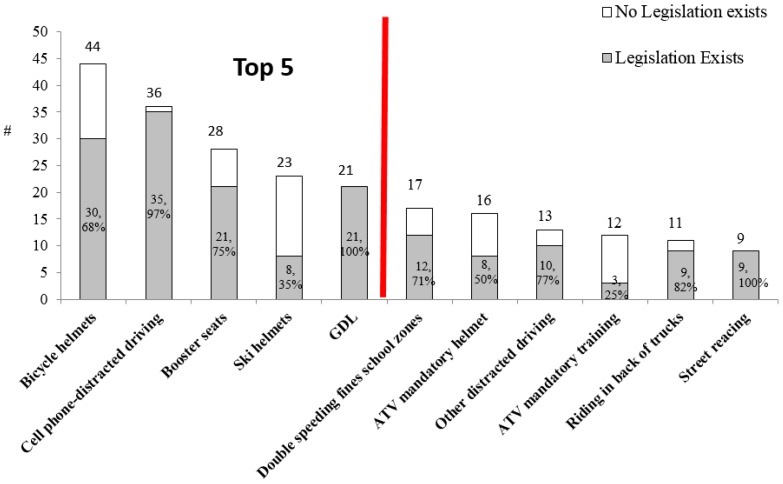
Top five topics and existing provincial legislation.

The top five injury legislation topics identified were: bicycle helmets, cell phone-distracted driving, booster seats, ski helmets, and graduated driver’s licensing (GDL, Figure 2). Although cell phone-distracted driving legislation targets adult drivers, it is also pertinent for children who are especially vulnerable as pedestrians and car passengers.

Table 1 portrays the provincial legislation implementation dates by topic. Only six respondents incorrectly indicated whether or not legislation existed; however; results were not influenced by these misclassifications as none of these respondents answered the subsequent questions related to their incorrect response.

There were 41 responses reporting barriers to legislation. The most common barriers to injury prevention legislation were competing policy priorities and insufficient managerial/political support/will. The most barrier responses were related to ski and bicycle helmet legislation (*n* = 15 each), as these lacked provincial legislation in several provinces.

**Figure 3:**
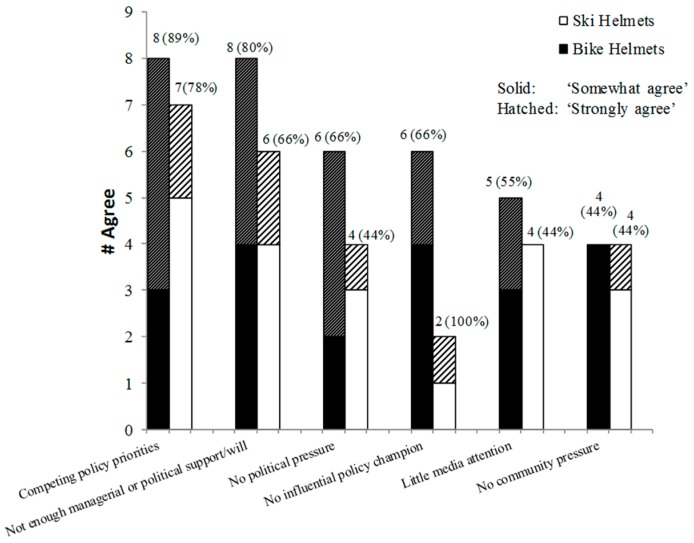
“Ski and bike helmet legislation has not been instituted in my province because….”

Despite both topics being helmet-related, there were differing barriers reported for each. For example, although 80% of respondents indicated insufficient managerial or political support/will and 66% reported no political pressure for bicycle helmet legislation, these were less frequently reported as barriers for ski helmet legislation (66% and 44% responses respectively, Figure 3).

**Figure 4:**
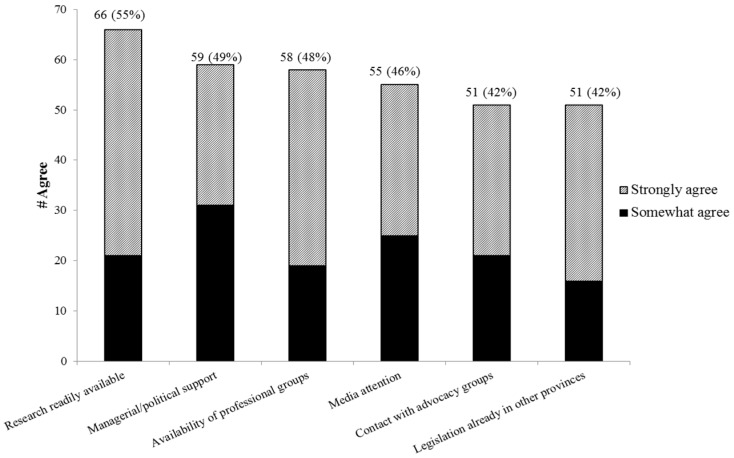
Legislation (all topics) as it currently stands has been instituted in my province because….

There were a total of 115 responses related to enablers of legislation (Figure 4). The most frequent enablers reported were, research/surveillance being readily available and the availability of managerial/political support and professional groups for consultation.

**Figure 5:**
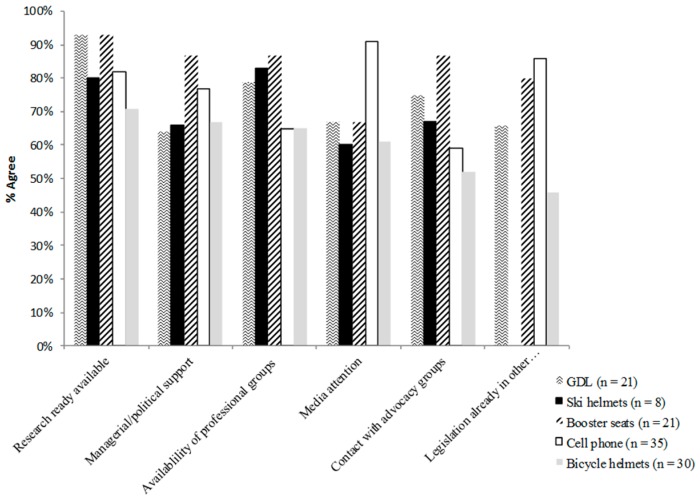
Legislation for (topic) __ as it currently stands has been instituted in my province because…* (* Somewhat/strongly agree have been summed together for clarity).

Figure 5 portrays the variability of enablers of legislation by injury topic. For example, a high proportion of respondents identified research as an enabler especially for GDL and booster seats, but research was not as important for ski and bicycle helmets. A high proportion of respondents identified media attention as an enabler for cellphone/distracted driving, but media was not reported as frequently for other topics. Legislation enacted in other provinces was especially important for cellphone/distracted driving and booster seats, but not as important for bicycle helmets and GDL.

One concern was that legislation more recently enacted may have different barriers/enablers than older legislation. However, the length of time since enactment was generally unrelated to specified enablers by topic. The exceptions to this were media attention and the existence of legislation already in other provinces. Agreement that these were enablers were reported by a high proportion of respondents in provinces with recently enacted legislation; booster seat in Saskatchewan and Manitoba (80%, media attention, 100% legislation in other provinces), bicycle helmet in Manitoba (100% and 100% respectively), and driving legislation in all provinces (91% and 86% respectively). Lower proportions of respondents indicated agreement that media attention and existence of legislation in other provinces were enablers in provinces with older legislation enacted >7 years previous to the survey; booster seat and bicycle helmet legislation in provinces other than Saskatchewan and Manitoba (60% and 70% respectively), bicycle helmets in provinces other than Manitoba (60% and 38% respectively) and GDL in all provinces (67% and 66% respectively).

There were eight emergent themes from the open-ended comments representing enablers and barriers:

(1) Population Burden and Severity of Injury

Respondent comments indicated that all identified injury topics could result in severe injuries. However, some stated that a low population burden could be a barrier to legislation:

“We have few if any serious brain or spinal cord injuries from this sport.” (PE)

“This legislation would only apply to a small portion of the population.” (SK) (*ski helmets*)

However, although numbers of injuries may be low, the severity of the potential injury was an enabler of legislation.

“Although the number of serious brain injuries is not numerous each year, the nature of the injury itself is severe enough to warrant action.” (NS) (*ski helmets*)

“I look at every head/brain as being precious and therefore any injuries due to not wearing a helmet are unacceptable. The long-term implications for a head injury on the individual, their family and society are great.” (ON) (*bicycle helmets*)

For other topics, both the numbers of injuries and the potential severity were recognized as enablers of legislation:

“Significant costs are associated with the number and extent of injures related to motor vehicle collisions involving new drivers.” (NS) (*GDL*)

“Over a third of the root cause *(of our provinces deaths and injuries on roads)* is driver distraction and while there are a number of distractions, cell phone use is a major one.”(SK) (*cellphone*)

(2) Evidence of Preventability, Risk Reduction

Lack or presence of strong evidence of the effectiveness of legislation in preventing injury was identified by respondents as an important barrier/enabler for some topics:

“Evidence of the actual impact is lacking and very difficult to generate, but we shouldn’t stop looking.” (ON) (*cellphone*)

“Research showed evidence that supported development of the legislation.” (ON) (*booster seats*)

“The evidence is incredibly strong for a positive impact reducing injuries and death.” (NS) (*GDL*)

“We know a lot about the mechanisms of injury and the role helmets play in protecting the face, skull and brain.” (ON) (*ski helmets*)

(3) Availability of Surveillance Data

The lack of surveillance system providing up-to-date data was reported as a barrier to the enactment of cellphone and bicycle helmet legislation:

“We know that distracted driving increases crash risk but we have little data on the number of crashes that are actually caused by cell phones … and even on how many drivers in BC use cell phones.” (BC) (*cellphones*)

“We don’t have a national, comprehensive injury surveillance system that I am aware of, and until we do, we are only “flying with one wing”.” (SK) (*bicycle helmets*)

Ski helmet use surveillance was identified as important in the enactment of legislation:

“…had been contracted to do helmet surveillance on the ski hills in years prior (*to the legislation*) along with qualitative research about why people were or were not wearing helmets.” (NS) (*ski helmets*)

(4) United Opinion Regarding Injury Prevention Topic

Some injury topics have distinct advocacy groups that support and oppose specific injury prevention policies, and this was seen as a barrier to the implementation of legislation.

“There was opposition from adults to requiring adults to use (bicycle) helmets … adults could make an informed choice. Adults were skilled riders unlike children …” (AB) (*bicycle helmets*)

“Bicycle helmets do reduce the likelihood of severe head injuries but I have mixed feelings about this issue. Other measures—better bike lanes/increased training of all drivers/*etc.* may be more effective. I also feel that the responsibility for preventing cyclist injuries falls road designers and on all road users and not just the cyclists.” (BC) (*bicycle helmets*)

“I live in a community with no alpine sporting venues. Ski resorts are used by people from all over. It is challenging to develop a collective voice when the population is relatively small and spread through (the province) and beyond.”(ON) (*ski helmets*)

The perception of the different applicability of some regulations in rural contexts was also identified as a barrier to legislation:

“There were arguments it should apply only to big cities as there are no options in rural locations.” (ON) (*GDL*)

“We believe that there is a fear of backlash from rural residents even though … a research study showed that rural residents know that their children are protected by the use of helmets.” (SK) (*bicycle helmets*)

Another barrier identified was that legislation could negatively impact physical activity:

“We want people to be active, and not limited through legislation in being so.“ (NS) (*ski helmet*)

“It would be nice to have a solid statement from a reputable source that deals with certainty about the rates of ridership.”(SK) (*bicycle helmets*)

Many respondents indicated that an enabler of legislation related to GDL, bicycle helmets and booster seats was a strong united voice regarding legislation specifically designed to protect children and youth which has less of a political risk:

“Keeping children safe from injury is an easy sell to the community at large and a good message to be associated with, politically.” (BC)

“Requiring bicycle helmets for persons under age 18 was seen as a low political risk option.”(AB) (*bicycle helmets*)

(5) Legislation Already Exists in Other Jurisdictions

The existence of legislation in other jurisdictions was seen to be an important enabler of legislation, particularly for booster seats and GDL:

“*My province* does not like to be first.” (PE) (*booster seats*)

“We looked at research and evidence from other provinces and components of their legislation.” (PE) (*GDL*)

(6) Timing

Respondents identified timing as important for increasing the profile of an injury topic and move legislation forward. The lack of a high profile case was identified as a potential barrier:

“I don’t think the profile is high enough. Unfortunately, it can take a high profile case where someone notable is injured to spark the debate and move forward policy decisions.” (ON)(*ski helmets*)

The personal experience and interest of politicians and the political context was instrumental to enacting legislation for bicycle helmets and ski helmets:

“…a friend of health minister was killed not wearing helmet.”(NS) (*bicycle helmets*)

“The latter two amendments … both came from opposition private members bills during periods where there was a minority government, eager to make deals with the opposition. These changes passed with all party support.” (NS) (*bicycle helmets*)

“Numerous other helmet laws were in place, the health minister at the time was interested in brain injury prevention. There was a majority government.”(NS) (*ski helmets*)

(7) Enforcement

Inadequate enforcement was a barrier to injury legislation as there was skepticism around whether legislation would be enforced with strict enough penalties, particularly for bicycle helmets.

“There is very little enforcement of the legislation that is currently in place for children under 18.” (ON) (*bicycle helmets*)

The availability of funds to support enforcement, as well as the small localized area of a ski hill, which is easier to enforce, was an enabler to ski helmet legislation:

“Some budget (*was available*) to add some supports to enforcement with legislation in *my province*, however, small ski hills easily enforced.” (NS)

“At a ski resort it should be fairly easy to enforce. No helmet, no ride.”(NS) (*ski helmets*)

(8) Costs of Equipment

Respondents also identified the costs of equipment as a potential barrier:

“Can everyone afford to purchase a helmet?”(NL) (*bicycle helmets*)

The availability of community group promotions or corporate donations to avert costs of equipment was an enabler of legislation.

“Initially, there were early promotions for free/low cost helmets for children and youth.” (ON) (*bicycle helmets*)

## 4. Discussion

This is the first study to describe barriers and enablers specific to child and youth injury prevention legislation in Canada. The most frequent barriers identified were competing policy priorities, followed by insufficient managerial/political support/will. The most frequent enablers identified were the availability of research/surveillance, managerial/political support and of professional groups for consultation. The findings emphasize the importance that strategies to implement evidence-based policies should focus on collaboration among research, management and the community.

Research availability was identified as an important enabler to enactment of injury legislation. Strong evidence of effectiveness of both the intervention and the legislation was important for all injury topics. The importance of surveillance data was emphasized, both to establish the injury rates due to a particular cause, and the prevalence of the use of protective devices (e.g., helmets).

Recent evidence has emphasized the need for researchers and policy makers to work actively together in the development of policy [11,12,13]. Morandi described how research can inform and evaluate public policy related to active living research in various jurisdictions in the United States [14]. Proposed ways that researchers and policy makers can work together include incentives, the use of knowledge brokers, organizational changes, the definition of research in a broader sense, and acknowledgment of the complexity of policy making [13]. In a systematic literature review, one of the most commonly reported barriers to the use of evidence for health policy was lack of relevance of research, and an important enabler was the inclusion of research summaries with policy recommendations [15]. The development of policy briefs by researchers has been proposed as an effective method to support evidence-informed policymaking [16].

Responses to the open-ended questions reflected the most frequent enablers to legislation identified in the survey questions; including timing, and the existence of legislation in other jurisdictions. However, several other important themes emerged from the open-ended questions that were not included in the survey; the important influence of a united opinion on the enactment of legislation particularly with respect to helmet laws, and of concerns regarding costs of protective equipment (booster seats and bicycle helmets) and adequate enforcement of the legislation (particularly for helmets). Particular attention to these issues for specific topics may facilitate the process towards the enactment of legislation.

Although there were common themes found among injury topics in this study, the results strongly highlighted the importance of considering injury prevention topics separately. Results indicated that there were different enablers and barriers depending on the topic. For example, media attention was identified as an enabler by a high proportion of respondents for cellphone/distracted driving, but not as frequently for the other topics. This may be a result of cellphone/distracted driving legislation being recent and that there is still current media attention related to this issue. Previous systematic reviews regarding the use of evidence for health and a wider range of policy topics, identified barriers and facilitators for all topics generally [9,15]. This study emphasized that important information may be lost if topics are considered together, and that attention to the uniqueness of issues surrounding particular injury topics may be instrumental in policy development.

The strengths of this study included the identification of barriers and enablers related to specific childhood injury topics. There was representation of policy makers from all provinces, so the results can be considered generalizable within the Canadian context; however, it is unknown if these results would apply to other countries. The limitations included the small numbers of respondents, which made it difficult to analyze each topic by province. Although the voluntary participation could be considered a source of participation bias, this was not considered an issue, as it was the intent of the study to gather information from injury prevention experts with vested interest in the topics, who would be the most likely to participate.

## 5. Conclusions

The findings of this study supported the importance of collaboration among researchers, politicians, and community members when developing strategies to implement evidence-based policies. Results also emphasized that different barriers and enablers to the enactment of legislation exist for different child and youth injury prevention topics. The consideration of the uniqueness of each topic may be essential to the successful development and enactment of injury prevention policy. Harmonization of evidence-based injury prevention policies is likely to reduce the burden of injury among children and youth in Canada, but the approach to enacting these policies will require unique strategies for each province and each topic.

## Figures and Tables

**Table 1:** Provincial legislation implementation dates (as of January 2015).

Province	Bicycle Helmets	Cell Phone-Distracted Driving	Booster Seats	Ski Helmets	Graduated Driver’s License
**British Columbia (BC)**	All ages, 1996	2010	2008	**X**	1998
**Alberta (AB)**	<18, 2002	2011	**X**	**X**	2003
**Saskatchewan (SK)**	**X**	2010	2014	**X**	2005
**Manitoba (MB)**	<18, 2013	2010	2012	**X**	2003
**Ontario (ON)**	<18, 1995	2009	2005	**X**	1994
**Quebec (QC)**	**X**	2008	2002	**X**	1997
**New Brunswick (NB)**	All ages, 1995	2011	2008	**X**	1996
**Nova Scotia (NS)**	All ages, 1997	2008	2007	2012	1994
**Prince Edward Island (PE)**	All ages, 2003	2010	2008	**X**	2000
**Newfoundland (NL)**	**X**	2010	2008	**X**	1999

**X**—No legislation.

## Appendix

### Case Study-Nova Scotia and Ski Helmet Legislation

In Nova Scotia the first all-ages mandatory snow sports helmet law in the world stating that only approved helmets can be worn (Bill No. 131), was enacted in November 2012. The four individuals who responded to the questions related to ski helmet legislation, identified themselves as policy makers, advocates, researchers and university instructors. All were satisfied with the existing legislation and did not think any changes were needed.

According to these respondents, it was not the number of traumatic brain injuries in Nova Scotia, but the severity of the injury and the visits to emergency departments that were the prime motivators for ski helmet legislation. Of the top overall six enablers identified for all injury topics in the survey (Figure 4), four were identified as enablers of ski helmet legislation; research readily available, managerial support and political will, availability of professional groups to consult with, and contact with advocacy groups.

Research was identified as an important enabler of this legislation, in terms of its availability, long-standing good relationships and collaboration with researchers, similar priorities of researchers and policy makers and that research was presented so that it was easy to understand and facilitated its use. It was emphasized that efforts were made to survey the current status of helmet use pre-legislation through active surveillance on ski hills, along with qualitative research.

Two of the other overall enablers identified in Figure 4, were not identified as being important by all respondents for the enactment of ski helmet legislation; media attention and legislation already existing in other provinces. It was stated that although there was not a significant amount of media attention related to ski helmets, the attention around the death of Natasha Richardson, an English actress, on a Canadian ski hill from a head injury in 2009, may have opened the opportunity to explore mandatory policy/legislation. There was no other province that had existing ski helmet legislation.

Several themes identified in the open-ended questions, were also reflected as important for ski helmet legislation; severity of injury, availability of surveillance data, enforcement and timing. It was noted that there was some initial start-up costs with the enforcement and with purchasing signs for the ski hills that they are required to display. Enforcement was incorporated into the duties of public health officers who travel around and enforce other pieces of public health legislation (e.g., tobacco, tanning beds, etc.). The timing of the legislation was also good, in that there were other helmet laws already in place in Nova Scotia, the health minister was interested in brain injury prevention and there was a majority government. There were also influential policy champions for this issue, both inside and out of government, including a group of neurosurgeons and the health minister.
